# Developing a Neuroimaging Biomarker for Amyotrophic Lateral Sclerosis: Multi-Center Data Sharing and the Road to a “Global Cohort”

**DOI:** 10.3389/fneur.2018.01055

**Published:** 2018-12-04

**Authors:** Robert Steinbach, Nayana Gaur, Beatrice Stubendorff, Otto W. Witte, Julian Grosskreutz

**Affiliations:** Hans Berger Department of Neurology, Jena University Hospital, Jena, Germany

**Keywords:** amyotrophic lateral sclerosis, MRI, data-sharing, NiSALS, quality-control, harmonization, multi-center

## Abstract

Neuroimaging in Amyotrophic Lateral Sclerosis (ALS) has steadily evolved from an academic exercise to a powerful clinical tool for detecting and following pathological change. Nevertheless, significant challenges need to be addressed for the translation of neuroimaging as a robust outcome-metric and biomarker in quality-of-care assessments and pharmaceutical trials. Studies have been limited by small sample sizes, poor replication, incomplete patient characterization, and substantial differences in data collection and processing. This has been further exacerbated by the substantial heterogeneity associated with ALS. Multi-center transnational collaborations are needed to address these methodological limitations and achieve representation of rare phenotypes. This review will use the example of the Neuroimaging Society in ALS (NiSALS) to discuss the set-up of a multi-center data sharing ecosystem and the flow of information between various stakeholders. NiSALS' founding objective was to establish best practices for the acquisition and processing of MRI data and establish a structure that allows continuous data sharing and therefore augments the ability to fully describe patients. The practical challenges associated with such a system, including quality control, legal, ethical, and logistical constraints, will be discussed, as will be recommendations for future collaborative endeavors. We posit that “global cohorts” of well-characterized sub-populations within the disease spectrum are needed to fully understand the complex interplay between neuroimaging and other clinical metrics used to study ALS.

## Introduction

### Acknowledging the Inherent Heterogeneity in ALS

It is widely accepted that amyotrophic lateral sclerosis (ALS) is a multifactorial disease, with an etiology that extends far beyond the selective vulnerability of motor neurons. Heterogeneity stemming from site-of- and age-at-onset, survival, genetic predictors, and the presence of frontotemporal dementia has severely constrained therapeutic translation (1). Precision biomarkers provide frameworks for early detection, tracking, and patient stratification, ensuring that treatment effects are not occluded by phenotypic variability. Today, neuroimaging in ALS isn't limited to merely structural-functional correlations and is on par with traditional “wet” biomarkers when it comes to group- and individual-level analyses (2, 3). Neuroimaging represents a crucial addition to the current repertoire of outcome metrics used in clinical trials; this includes respiratory measures, muscle strength, and the Revised Amyotrophic Functional Rating Scale (ALSFRS-R), the ambiguity of which has been previously reported (4).

### NiSALS: Why Data-Sharing Is the Way Forward for ALS Research

Given the underlying complexity, low prevalence, and poor patient longevity, larger, multi-layered data sets are needed to capture the full spectrum of pathological signatures in ALS and develop population-specific markers. Such data sets can only be generated through well co-ordinated, multi-center efforts. In the wider neurodegenerative field, ventures like the Alzheimer's Disease Neuroimaging Initiative (ADNI) have demonstrated the analytical power of transnational collaborations. ADNI was launched in 2004 as a multi-site, longitudinal study to develop biomarkers for Alzheimer's Disease. To date, over 1,700 publications spanning several topics have resulted from ADNI data (5–7). ADNI has inspired similar initiatives in various neurodegenerative conditions, including ALS. “Sampling and Biomarker Optimization in ALS and other Motor Neuron Diseases” (SOPHIA) was the most comprehensive of these efforts and ran from 2012 to 2016, with ~2.4 million EUR in funding (http://www.neurodegenerationresearch.eu/fileadmin/Project_Fact_Sheets/PDFs/Biomarkers/SOPHIA_Fact_Sheet.pdf). It was conceived with the goal of harmonizing optimal methodologies for biomarker identification, thereby providing a pan-European framework within which existing and future endeavors could integrate. By consolidating expertise from over 15 leading European centers, SOPHIA helped establish the Progeny database: a web-based sampling infrastructure for the streamlined collection of clinical, neurophysiological, imaging, and bio sample-based data. Furthermore, the development of a centralized repository system for MRI data as part of SOPHIA led to the establishment of The Neuroimaging Society in Amyotrophic Lateral Sclerosis (NiSALS). The first NiSALS meeting (Oxford 2010) recognized the need for quality-controlled and harmonized MRI data and led to the publication of consensus guidelines on data acquisition (8). Annual meetings have since cemented NiSALS' role as an international consortium fostering neuroimaging as a key tool for understanding ALS. Today, a growing number of centers across Europe, North America, and Australia are NiSALS members, and are actively contributing data and hosting symposiums.

Each year has brought its own set of hurdles and achievements, showing that large-scale efforts like NiSALS rather than being monolithic, have the capacity to continuously adjust to the needs of the scientific community (9, 10). This review, while not exhaustive, will use NiSALS to illustrate the stakeholders and processes involved in multi-center data sharing. We hope to demonstrate that the associated challenges, while not insignificant, are surmountable, and that only global cohorts can generate the volume and variety of data needed to understand complex disorders like ALS.

## The NiSALS Ecosystem: A General Overview

**Figure 1:**
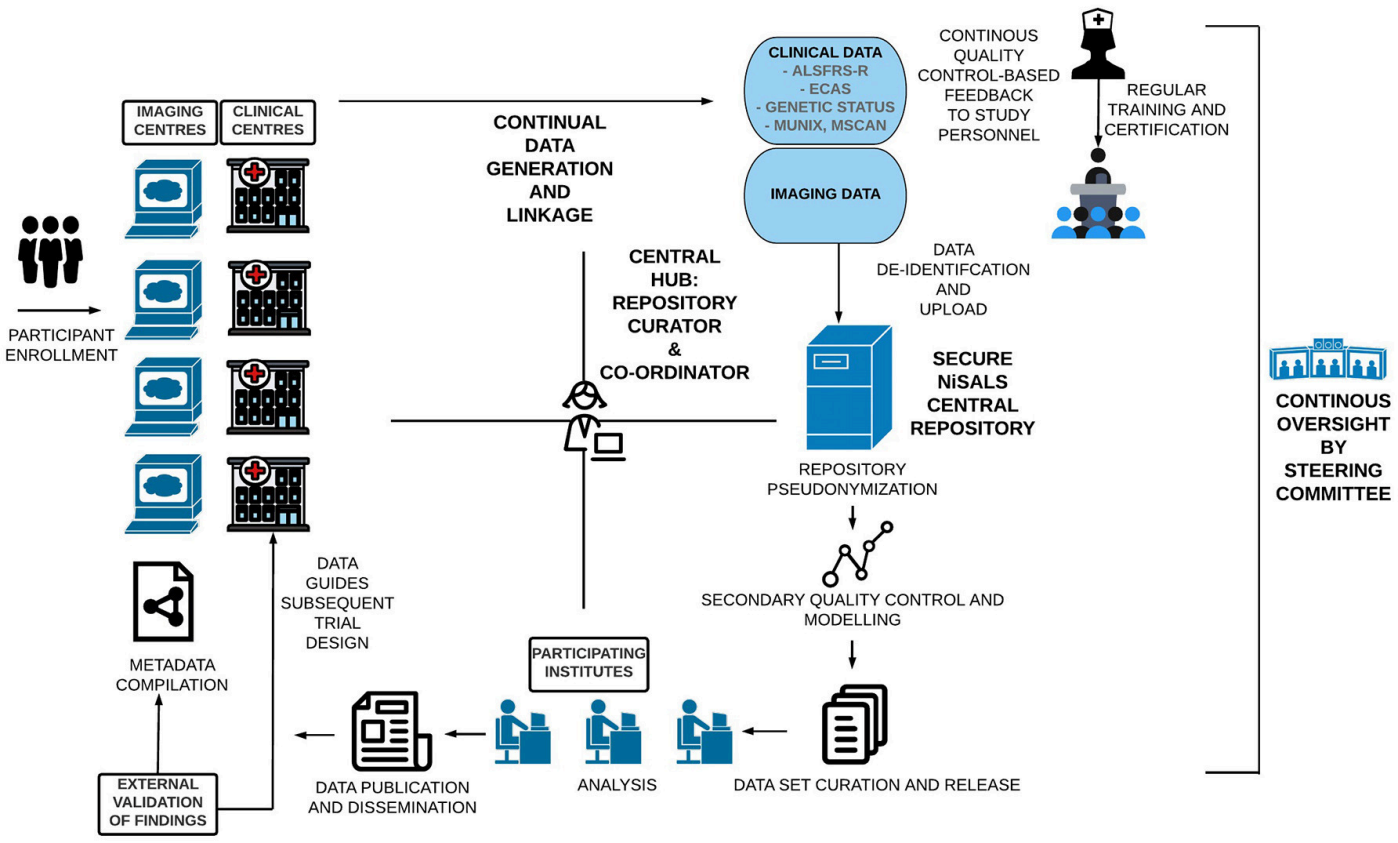
Visual Schematic of the NiSALS ecosystem, including representations of future recommendations.

NiSALS' primary goal was always to function as a self-sustaining entity that provides the ALS community with the tools needed to advance neuroimaging-based research. The establishment of a secure central repository and the institution of a formally elected steering committee (http://nisals.net/?page_id=159) were significant first steps. The committee is responsible for the democratic governance of NiSALS activities, including making timely project and data-transfer decisions, event management, and liaising with third-party stakeholders. The general flow of data and stakeholder-interactions is depicted in Figure 1. Participating centers can continuously upload MRI data into a designated repository slot. Folders are available for the collection of additional clinical data that can be integrated into the server architecture for appropriate dissemination. However, individual centers are responsible for (a) obtaining approval for data sharing from the relevant ethics committee or review board and (b) appropriate data coding. Contributing centers are provided with guides, accessible through the NiSALS webpage, that include recommended packages of established freeware imaging resources to ensure thorough data de-identification prior to upload. The uploaded data then undergoes an additional round of pseudonymization (discussed in Section Data De-identification) for complete legal compliance. Crucially, each center has exclusive read/write access to their own data, in addition to having read-only access to common information areas. The repository creates individual data root trees to prevent users from accessing data domains that aren't theirs. The exact repository content for each contributor is kept confidential to add credence to the NiSALS curation mechanisms.

Figure 1 shows that the centralized communication hub (overseen by the NiSALS co-ordinator and repository curator) is essential for the streamlined running of the platform. Given the dynamic data sharing that NiSALS entails, the hub serves as a liaison point for all stakeholders, especially since data generators have expressed a desire for continuous feedback on data content and usage. The co-ordinator is also responsible for organizing annual NiSALS meetings and collection of associated materials.

The NiSALS webpage (https://nisals.net/) is an indispensable platform tool. It is used for administrative duties, including member and event management, compiling support documentation, and regularly updating legal compliance notices. The website also serves as an entry-point for interested stakeholders, and is crucial for bolstering outreach. In the future, the webpage will contain teaching materials and enable center-specific repository content viewing.

## Legal Frameworks and Data-Sharing

Central to any data-sharing effort is the cultivation of trust. All data-handling procedures are therefore in accordance with the NiSALS bylaws, which are designed to be collaborative and transparent. The bylaws recognize that all users need to be treated equally and should shoulder both the costs and benefits associated with embargo-free data sharing. Data sharing within NiSALS most closely resembles the “learned intermediary” model (11). Briefly, the model stipulates that an independent panel reviews applications and grants access to data primarily on the basis of applicant expertise and the quality of the proposed research. Within NiSALS, all applications are reviewed by the steering committee. Applicants must clearly detail (1) intended scientific analyses, (2) expected time-line to completion, and (3) specifications of required data in a project proposal. Successful applicants are bound by a stringent data-sharing agreement i.e., a legal mechanism to enforce NiSALS' core bylaws. Of note, are the following specifics:

1. Following publication, the released data set has to be destroyed
2. The released data set cannot be shared with third parties
3. Any additional analyses must first be vetted by the aforementioned application process.

Crucially, NiSALS recognizes that ownership of uploaded data permanently resides with the uploading center, regardless of which stage in the data-handling cycle the data is at. Thus, contributors also have the right to have their data removed from the repository upon written request.

As with any scientific undertaking, there arises the question of publications. NiSALS encourages collaborators to define and agree in writing to authorship roles prior to project commencement. Authorship credit should be in keeping with the guidelines developed by the International Committee of Medical Journal Editors. Responsible data generators should be offered contributory roles, regardless of the volume of data used. Finally, authors must reference NiSALS in resulting publications.

In summary, NiSALS operates with maximum practicability to ensure that (a) the immense benefits of sharing data outweigh the potential risks and (b) there is no disproportionate burden on data generators. Of note, when working with multiple stakeholders across geographical locations, it is unlikely that a “one size fits all” data-sharing agreement can be developed, as the judiciary requirements vastly vary between and within countries and institutions. Similar repositories should ensure that while their legal frameworks are exacting, they should be broad enough to facilitate the desired results.

## Data De-Identification

As within other research domains, data sharing within neuroimaging is a constant balance between protecting confidentiality and sharing information to facilitate in-depth analyses. Multi-centre initiatives add further complexity, as individuals have to be universally identifiable, with seamless linkage of their participation across various projects.

Substantial efforts in bolstering technical inter-operability in diagnostic imaging resulted in the establishment of the “Digital Imaging and Communications in Medicine” (DICOM) format. NiSALS adopted it for repository uploads, as the image-headers specify the parameters used during image acquisition. This information is needed for subsequent quality-control (QC) and harmonization procedures as it is essential for determining which parameters are most likely to have disturbed image quality or be most relevant during multi-center data comparison. However, all original DICOM-files also contain information that needs to be safeguarded to maintain participant confidentiality. De-identification within NiSALS is conducted in two basic steps explained below.

### Basic DICOM Pseudonymization

DICOM files are first pseudonymized by removing information linked to participant identity. As mentioned above, individual contributing centers are responsible for ensuring this prior to uploading data. Further, private DICOM-header fields that are modality- and vendor-dependent must be removed (12, 13). NiSALS' internal naming conventions require that all uploaded files use local center-specific pseudonyms; this allows contributors to (a) keep track of uploaded data, (b) continuously provide additional data sets, and **(c)** link insights from the analysis process back to the original data set.

### Internal Repository Pseudonymization

Data within the repository are also subjected to secondary internal checks prior to being released for analyses. These checks include the removal of identifiable facial structures (defacing) and auxiliary whole-DICOM header de-identification (14). The latter is always in keeping with the current recommendations by the National Electrical Manufacturers Association that regularly lists relevant public header fields (15). Any center-specific information is implicitly removed, as researchers using the data should be blinded to its source of origin. All study participants are allocated a unique NiSALS-generated internal pseudonym. As centers subsequently submit associated data, it is essential to maintain linkage through these layers. Therefore, NiSALS' requires all additional data to be submitted to the repository following the same pipeline of pseudonym generation, thus allowing integration with the individual participant.

## Quality Control Procedures

As a first layer of QC, robust mechanisms are needed to prevent inclusion of corrupted MRI data in subsequent analyses. While being susceptible to obvious errors (e.g., extinction-artifacts), images in a multi-center set-up can also be compromised by scanner-hardware/software and modality-specific factors that may result in bias further downstream (16–18). Manual analysis and exclusion/inclusion of data sets by a trained rater is time- and labor-intensive, and contingent on rater expertise. Conversely, while automated QC procedures may overcome inter- and intra-rater variability, their applicability to one distinct data-set may not necessarily be transferable to new data acquired from different sites, thereby still necessitating visual checks by human operators (19).

Contributing centers are also responsible for complying with initial QC requirements prior to upload to minimize the risk of corrupted data entering the repository. Subsequently, modality- and analysis-specific QC approaches are applied to the stored data. Here, the challenge lies in identifying artefacts and correcting for scanner-specific factors prior to the data entering a multi-center analysis, whilst minimizing time expenditure and potential manual bias.

**Figure 2:**
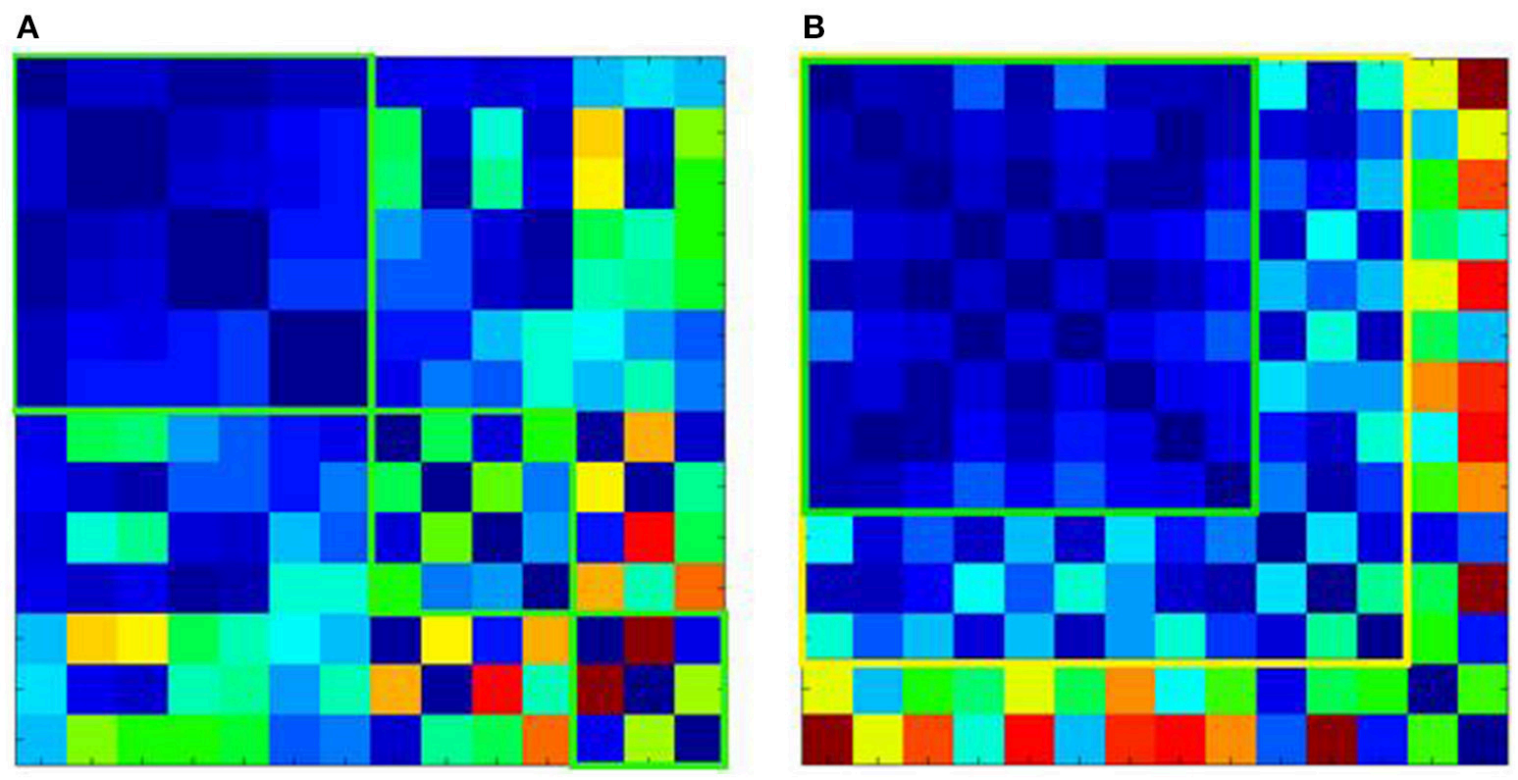
Mahalanobis distance analysis of quality parameters for T1-weighted images of 14 ALS centers. **(A)** Shows the Mahalanobis distances of the raw T1 data, revealing 3 clusters of centers, which although internally homogenous (green squares) could not be pooled into one large data set. **(B)** Shows the effect of preprocessing. This allows pooling of T1 data from additional centers with good (green square) or acceptable homogeneity (yellow square). However, 2 centers although homogenous with each other, could not be pooled with the other data sets (shown in the last 2 rows or right-most columns, respectively).

QC mechanisms that enable the processing of large multi-site data sets have been developed for T1 data. First, covariance screening of image parameters related to inhomogeneity or noise is conducted for outlier identification. Hereafter, software-based preprocessing algorithms for raw T1 images (e.g., as available with SPM; https://www.fil.ion.ucl.ac.uk/spm/) facilitate correction of scanner- and protocol-induced systematic artefacts, whilst minimizing alteration of disease-specific signatures. Mathematical models like Mahalanobis distance analysis can help minimize and eliminate software-bias and overcorrection when identifying technical artefacts in multi-center data sets. These models provide a meta-analysis of image quality parameters, indicating which data sets are similar and amenable to pooling as illustrated in Figure 2. Ultimately, all algorithmic solutions involve compromise between correction and the biological signal and therefore need to be continuously improved with feedback from all users, which is naturally extremely resource-intensive.

Similar QC procedures have been adopted by the NiSALS DTI Study Group; these include the automated exclusion of particular gradient directions in single DTI data sets (20, 21) and correction for acquisition-derived eddy-current-induced geometric distortions (20). The NiSALS DTI Study Group used these QC procedures to correct 442 single DTI data sets (from 8 contributing ALS centers) for artefacts like susceptibility-induced geometric warping, participant motion and chemical shift, prior to further analysis (10).

## Cross-Platform MRI Interpretation and Harmonization

As discussed above, multi-center-studies suffer from poor data comparability, owing to scanner-hardware/software differences. For instance, a study using MRI scans of the same subjects taken at different sites showed that DTI-derived values (e.g., fractional anisotropy) showed moderate reproducibility between different scanners, while particular higher field strengths and enlarged acquisition resolutions decreased inter-center variability (22).

Even if different sites use identical scanners, variance can still arise from differences in derived structural and functional imaging information; however, harmonization can improve comparability (23, 24). Processing procedures used at different sites can also contribute to variability. Therefore, as with ADNI, standardized MRI-data sets that rely on harmonized acquisition schemes and have undergone QC are needed to support direct comparisons of different processing methods.

The majority of MRI-centric publications in ALS are offset by low sample sizes and high phenotypic heterogeneity within disease cohorts (3). One of NiSALS' core objectives was to define rules for MRI acquisition to help maximize accuracy and comparability and thereby enlarge study sample sizes. The published consensus guidelines therefore detail essential and desirable recommendations for T1, DTI, functional MRI and spectroscopy data acquisition (8).

ADNI uses a cross-platform calibration procedure that utilizes traveling phantoms for data harmonization (25). Certainly, implementing a comparable procedure for ALS centers on a global scale would require a substantially larger investment of financial and human resources, partly due to the lower prevalence of ALS (26). Therefore, NiSALS' current harmonization efforts focus on (a) ensuring that previously acquired neuroimaging data meets standards for multi-center analyses and (b) using feedback to maximize acquisition accuracy. Ultimately, MRI acquisition, and harmonization protocols need to be diligently updated to reflect the latest technical advances.

Although current uploads primarily include DTI and T1 data (~1,000 participants for the latter), NiSALS welcomes the integration of multi-modal imaging techniques and combined structural-functional approaches and hopes to collect and disseminate data that reflects the full breadth of neuroimaging methods currently available. However, appropriate set-ups for the acquisition and use of these modalities also need to be concurrently developed if they are to be used for multi-site projects (9).

## Clinical Data Linkage

Owing to its complexity, ALS cannot be studied as a homogenous disease. In-depth multi-modal data are required for the classification of clinical, neuropsychological and imaging-based phenotypes of sporadic disease and genetic variants. This is particularly relevant when developing neuroimaging biomarkers. Incomplete patient characterization has limited several neuroimaging-based studies; the lack of clinical data constrains both accurate distinction of ALS from disease mimics and understanding of pathophysiology and progression. To fully understand the degree to which MRI and other modalities can assess disease activity and quantitate functional progression, they have to be placed within the framework of core clinical data and other biomarkers. The latter is crucial as individual biomarkers display different patterns across the disease course and in different clinical phenotypes; this has been well described for Alzheimer's Disease (27).

Naturally, this is contingent on available resources and NiSALS therefore advises contributing centers on clinical data to submit alongside MRI data sets; these have been previously published (https://www.encals.eu/wp-content/uploads/2016/09/2015-01-14-ALS-Core-clinical-dataset.pdf). In particular, NiSALS recognizes the importance of genotyping individuals and studying mutation carriers in presymptomatic disease phases to understand how genetic factors may influence the behavior of different markers (9).

Further, although data from healthy and disease controls is being continuously uploaded to the repository and requested in project proposals, both NiSALS and future efforts need to rigorously tackle the lack of longitudinal data from these subjects.

Although a detailed consideration of disease progression models is beyond the scope of this review, these are important tools for describing the disease course, particularly when clinical data cannot be collected at regular time-points for patients. Models can also help identify center-dependent and independent biological components. For instance, the newly developed D50 model enables random sampling of patients, comparisons between different progressor types and the placement of biomarker profiles within the functional time course of patients (28, 29).

## Future Directions and Conclusions

ALS, although highly heterogeneous, has the advantage of being measurably progressive. It is crucial to tap into neuroimaging's potential and use quantitative tools like MRI to describe the disease and understand its spread. Efforts like NiSALS can help the community develop and execute high-level data sharing, facilitate the replication of results and avoid unnecessary duplication of efforts. The ecosystem described here provides a structure for continuous QC and feedback that can help identify markers that are readily transferable to both the clinic and industry. Indeed, NiSALS hopes to establish well-defined collaborations with industrial partners looking to develop neuroimaging as an outcome metric for clinical trials. NiSALS can also offer its experience in implementing best practices, efficiently executing research, and disseminating results for the benefit of the neurodegenerative community. Future efforts must build on this momentum and endeavor to make the exercise more patient-centric by boosting engagement with them and communicating scientific results to them and the lay population. Stakeholders should also consider collecting meta-data on the outcomes of data sharing and how the process can be modified to better serve the community's needs.

Resources must also be directed toward building comprehensive, well-characterized multimodal biomarker panels. These can help expand the role of imaging beyond reductive clinico-structural correlations to a precision tool that can capture subtle pathological changes in population and individual-level analyses.

## Author Contributions

All authors listed have made a substantial, direct and intellectual contribution to the work, and approved it for publication.

### Conflict of Interest Statement

The authors declare that the research was conducted in the absence of any commercial or financial relationships that could be construed as a potential conflict of interest.

## Abbreviations

ADNI: Alzheimer's Disease Neuroimaging Initiative
ALS: Amyotrophic Lateral Sclerosis
ALSFRS-R: Amyotrophic Lateral Sclerosis Functional Rating Scale Revised
DICOM: Digital Imaging and Communications in Medicine
DTI: Diffusion Tensor Imaging
MRI: Magnetic Resonance Imaging
NiSALS: Neuroimaging Society in Amyotrophic Lateral Sclerosis
QC: Quality Control
SOPHIA: Sampling and Biomarker Optimization in ALS and other Motor Neuron Diseases.
